# ‘I am very glad and cheered when I hear the flute’: The Treatment of Criminal Lunatics in Late Victorian Broadmoor

**DOI:** 10.1017/mdh.2016.56

**Published:** 2016-10

**Authors:** Jade Shepherd

**Affiliations:** School of History, Arts Two, Queen Mary University of London, Mile End Road, London, UK

**Keywords:** Broadmoor, Criminal Lunatics, History from Below, Moral Treatment, Psychiatric Pessimism

## Abstract

Through an examination of previously unseen archival records, including patients’ letters, this article examines the treatment and experiences of patients in late Victorian Broadmoor Criminal Lunatic Asylum and stakes the place of this institution within the broader history of therapeutic regimes in British asylums. Two main arguments are put forth. The first relates to the evolution of treatment in Victorian asylums. Historians tend to agree that in the 1860s and 1870s ‘psychiatric pessimism’ took hold, as the optimism that had accompanied the growth of moral treatment, along with its promise of a cure for insanity, abated. It has hitherto been taken for granted that all asylums reflected this change. I question this assumption by showing that Broadmoor did not sit neatly within this framework. Rather, the continued emphasis on work, leisure and kindness privileged at this institution into the late Victorian period was often welcomed positively by patients and physicians alike. Second, I show that, in Broadmoor’s case, moral treatment was determined not so much by the distinction between the sexes as the two different classes of patients – Queen’s pleasure patients and insane convicts – in the asylum. This distinction between patients not only led to different modes of treatment within Broadmoor, but had an impact on patients’ asylum experiences. The privileged access to patients’ letters that the Broadmoor records provide not only offers a new perspective on the evolution of treatment in Victorian asylums, but also reveals the rarely accessible views of asylum patients and their families on asylum care.

In August 1883, Matthew Jackson Hunter, a patient at Broadmoor Criminal Lunatic Asylum, wrote to his sister:

It is a splendid block of buildings… has an extensive view and is very healthy… the patients spend most of their time… exercising in the gardens, reading the daily papers, monthly periodicals etc., there is also a well selected library… a cricket club, billiards, cards and other amusements. In the wintertime we have entertainments given by the patients, such as plays, singing, etc. [We] have a good brass band which gives selections of music every Monday evening during the summer months on the terrace opposite the chapel… [We] are treat[ed] with kindness by the officials placed over us, [and] have free conversation among the other patients.^1^

Scholarship charting the transition from therapeutic positivism to psychiatric pessimism within nineteenth-century psychiatry suggests that, when Hunter was writing, Victorian asylums were shrouded in pessimism: authoritarian superintendents, who had abandoned all hope of curing insanity, ruled over-populated institutions within which patients were forced to endure a life sentence.^2^ Hunter’s testimony does not sit comfortably within this narrative, and he was not alone in his positive representation of Broadmoor.

Twenty years before Hunter wrote this letter, Broadmoor, the first criminal lunatic asylum in England and Wales, opened its doors. The opening of the asylum followed decades of unease regarding the proper treatment of criminal lunatics: individuals who had committed crimes while insane, or who had developed insanity in prison. The early to mid- nineteenth century saw much debate concerning provision for criminal lunatics, most of whom were held in county asylums and prisons.^3^ Concern intensified in the 1850s when rising patient numbers, and increasing concern about the association of criminal lunatics with ordinary lunatics, came to a head.^4^ At Bethlem, all criminal lunatics were confined together regardless of offence or mental condition and some physicians expressed concern. Bethlem’s superintendent, W.D. Hood, believed this practice disturbed the discipline and treatment of ordinary lunatics.^5^ Eminent alienist, John Charles Bucknill, expressed concern that the wretched condition of Bethlem’s galleries and yards meant that treatment was impossible.^6^ The Association of Medical Officers of Asylums and Hospitals for the Insane, to which Bucknill belonged, called for the removal of criminal lunatics from Bethlem, a place judged so tedious that it inspired them to quote Italian poet, Dante: ‘Who enters here must leave all hope behind.’^7^ They wished to see in England a criminal lunatic asylum similar to that in Dundrum, Ireland, which opened in 1850. Dundrum’s patients were surrounded by acres of land, worked at their trades and were provided with various amusements.^8^

In 1857, the government announced plans to build Broadmoor and, in 1860, the Criminal Lunatics Bill was drawn up to make ‘better provision for the custody and care of criminal lunatics’.^9^ The Bill provided for the Lunacy Commissioners’ annual inspection of Broadmoor, and it empowered the Home Secretary to appoint a Council of Supervision to manage the asylum and the treatment of patients. Under the Council reigned the superintendent whose job it was to ‘superintend… the medical and moral treatment of the asylum’. He controlled all other officers, attendants and servants.^10^ Broadmoor opened to ninety-eight female patients in 1863 and to 221 male patients in 1864. There was one female block with space for one hundred patients and six male blocks with space for 400 patients.^11^ There were two classes of patient: Queen’s pleasure patients, who had been found insane before or during their trial; and insane convicts, who had become insane while undergoing a term of penal servitude and were transferred to Broadmoor from prison until their sentences expired and they were discharged to another asylum or released, or they were declared sane and sent back to prison until their sentences expired. Broadmoor was run like any ordinary asylum, and its patients were treated like ordinary patients – it did not matter that they had committed crimes.^12^

Scholarly work on Broadmoor has hitherto tended to focus on the asylum’s establishment and architecture and on the published works of its superintendents and, while there is much scholarship detailing the crimes and trials of individuals who were found insane and committed into Broadmoor, patients’ experiences inside the asylum have not been thoroughly examined.^13^ The opening of the Broadmoor archive in 2008 revealed sources that help to shed new light on Broadmoor and the experience of its patients. This article is based upon an examination of this archive: annual reports, minutes of meetings, correspondence between the asylum and the Home Office and patient case files. The latter included medical reports, brief notes and memorandums written by the asylum’s attendants, usually to the superintendent, and letters to and from patients, patients’ relatives and friends and the authorities. Approximately 550 patient case files were examined, each of which contained the documents just outlined, but in varying numbers; some, for instance, contained two letters, others over one hundred.

Following Roy Porter’s call for a history of madness from below, historians have undertaken much wonderful research into the social history of madness and asylums, including the patient experience.^14^ Alongside art, medical diaries, autobiographies, manifestos and ‘asylum scribbles’, patients’ letters have been instrumental for numerous scholars when examining what patients and their families felt about the asylum and insanity.^15^ As Jonathan Andrews has suggested, when using personal correspondence to reconstruct beliefs and experiences, we need to be aware of ‘sins of omission’, bias, censorship and the intended audience.^16^ Nevertheless, patients’ letters are valuable because they allow us to hear from the patient directly, rather than through physicians’ descriptions.^17^ Although Marjorie Levine-Clark has noted that ‘the thorny question of how we hear a patient’s voice… is especially difficult with the insane, whose rational ability to represent themselves is an issue’, patients’ letters should not be dismissed as meaningless ramblings.^18^ Broadmoor patients’ letters are often intelligible and articulate accounts of asylum life.^19^ There were patients in Broadmoor who, according to medical testimony, had lost all reason, but there were also two other types of patient who wrote letters: those who were deemed only partially insane and those who were sane but refused discharge because they did not have anyone willing or able to care for them, or because their crime was so heinous that the authorities were waiting for a period of time to pass before they sanctioned their discharge.^20^

Accessing patients’ letters is notoriously difficult. They rarely survive in the archive, and those that do remain tend to have been written by the middle- and upper-class patients.^21^ Moreover, because some asylum authorities tended to retain letters that portrayed the asylum negatively, treating them as demonstrative of a patient’s insanity, we are left with a disproportionately bleak portrayal of asylum life.^22^ The Broadmoor archive is unusual in three ways: first, the existence of patients’ correspondence marks it out from some other depositaries; second, the majority of the asylum’s patients belonged to the working classes, a vast group whose asylum and life experiences tend to be otherwise less documented; third, in contrast to what happened at some other asylums, Broadmoor’s superintendents retained letters displaying both positive and negative responses to the asylum. In the case of positive depictions, copies were sometimes made before the original was posted. While negative representations might have been retained as a demonstration of patients’ mental state or cooperation with treatment, letters in which patients or their friends and family praised the care and treatment received at Broadmoor might have been kept because they could be used in official reports and publications to promote the institution. As it is impossible to establish what proportion of positive and negative letters were retained, and important to remember that illiteracy prevented some patients from recording their thoughts, the letters do not lend themselves to a systematic assessment of patient satisfaction or lay opinion; but that is not the intention here.^23^ Certainly, some patients and their families objected to confinement but, while some of the letters included in this article represented Broadmoor negatively, the majority are positive reflections of, and responses to, the asylum. It is not possible to know how widely such opinions were held, but the letters utilised here show us how *some* individuals represented asylum life, and they help to qualify the gloomy representations of the late Victorian asylum that historians have constructed based upon patients’ (negative) accounts.

I examine the treatment of Queen’s pleasure patients and insane convicts at Broadmoor between 1863 and 1900, staking a place for the asylum within the broader history of regimes of treatment in British asylums. In the process of examining treatment at Broadmoor, the article lends support to scholars such as Anne Digby and Nancy Tomes who have questioned Michel Foucault’s contention that moral treatment functioned as a form of social control.^24^ I put forth two main arguments. The first relates to the evolution of treatment in Victorian asylums, re-examining the broad assumption that psychiatric pessimism affected all asylums simultaneously. Historians tend to agree that in the 1860s and 1870s psychiatric pessimism took hold, as the optimism that had accompanied the growth of moral treatment along with its promise of a cure for insanity abated. Moreover, historians tend to write in general terms when describing psychiatric pessimism: it was this ‘spectre of chronicity, this horde of the hopeless, which… dominate[d] Victorian psychiatric theorizing and practice’.^25^ An examination of the Broadmoor sources, patients’ letters especially, helps to enrich understandings of the transition from positivism to psychiatric pessimism within Victorian asylums. I argue that Broadmoor does not sit neatly within the current framework and that, when pessimism eventually crept into the regime, it was not all encompassing. Second, I show that, in Broadmoor’s case, moral treatment was determined not so much by the distinction between the sexes as the two different classes of patient – Queen’s pleasure patients and insane convicts – in the asylum. This distinction between patients not only led to different modes of treatment within Broadmoor, but had an impact on patients’ asylum experiences.^26^

## 1 Treating Queen’s Pleasure Patients

Harking back to the views of early nineteenth-century alienists and asylum physicians, the Lunacy Commissioners warned against the use of restraint and seclusion at Broadmoor because they believed it had a detrimental effect on patients’ minds.^27^ According to moral managers such as John Conolly, the success of non-restraint was dependent upon the character and behaviour of the attendants.^28^ Broadmoor’s *Rules* stated the importance of staff through the adoption of the language and approach of earlier advocates of moral treatment: ‘Kindness and forbearance are the first principles in the care and management of persons of unsound mind; few such persons are beyond their influence.’^29^ In March 1863, Reverend Burt, who had previously worked at Hanwell asylum with Conolly, was appointed chaplain. Burt drew upon his observations at Hanwell ‘that the moral and religious improvement of lunatics is to be aimed at… by the unceasing influence of the attendants’.^30^ He recommended ‘every measure tending to promote morals and religion’ among the attendants should be taken, and a library and reading room to teach them morality and values subsequently opened.^31^ Burt also supervised the secular teaching of patients and took charge of religious instruction, an important element of treatment.^32^ He delivered Church of England services and led daily prayers.^33^ Patients could also attend Sunday services alongside Broadmoor’s staff and their families. Based on his observations of female patients, Burt recorded that there was ‘evidence that even criminal lunatics are susceptible to religious impressions’.^34^ Burt’s interest in both staff and patients highlights the dual moralising mission to which he ascribed.

In 1873, superintendent William Orange [1870–86] declared: ‘It cannot be lost sight of that one object with which [patients] have been sent to this asylum, is that they might receive such treatment as may be calculated to promote the recovery of their mental health.’^35^ This view prevailed for the next twenty-three years, during which time patients were provided with various leisure activities and amusements ‘recognised as a valuable and necessary means of treatment for the insane’.^36^ Periodicals, newspapers and journals were provided, as were books, which Burt described as being ‘of great moral value; they afford mental occupation to a considerable number of all classes of patients, and both amuse and instruct them during many hours which, without this humane provision, would be spent in weariness, in bitter reflection, or in angry discontent’.^37^ Patients were given singing lessons and taught how to play instruments, and some joined the asylum band.^38^ The band entertained patients, as did exhibitions of the magic lantern and theatrical performances put on by Broadmoor’s staff. Patients played games, including cards, dominoes, billiards and chess and, in the summer months, male patients played croquet and cricket. There was also ‘ample provision for walking exercise’ and female patients could take drives into the neighbouring country.^39^

Early nineteenth-century alienists deemed an asylum’s location, architecture and interior design an important element of treatment.^40^ In 1859, the Lunacy Commissioners declared that Broadmoor should ‘have a general character of a hospital, and should be constructed as to have a cheerful aspect both within and without, and not to suggest the idea of custody or confinement’.^41^ In 1867, they recommended surrounding patients with ‘cheerful objects’ (artwork and plants) to promote recovery; they continued to endorse bright surroundings into the late 1890s.^42^ Similar to some county asylums where patients were categorised and separated according to sex and social class, at Broadmoor, there were numerous accommodation blocks, including separate blocks for women.^43^ Patients were also separated according to the threat they posed and male patients according to social class. Block one housed dangerous and disruptive male patients and block two housed male patients who had received a ‘superior’ education or had ‘been accustomed to greater comforts than general patients’.^44^ Patients who misbehaved were transferred to block one so that they could not disrupt treatment.^45^

The asylum authorities acknowledged that Broadmoor marked an enormous improvement on Bethlem. In his reports for both 1863 and 1864, superintendent John Meyer [1863–70] noted that ‘many patients have improved… since their admission’, something he attributed to their new, improved, surroundings.^46^ The Lunacy Commissioners reported that Bethlem’s former patients spoke ‘of the change in their condition for which they are extremely grateful’.^47^ Of course, such positive observations might have served the authorities’ interests – justifying the existence and cost of the institution^48^ – but it is notable that some patients, under superintendents Meyer, Orange and David Nicolson [1886–96], also commented positively upon Broadmoor’s regime and staff in their correspondence.^49^ Some detailed Broadmoor’s numerous leisure activities to their families. In response, the brother of patient Lucy Thompson told her: ‘We are both very pleased that you made yourself happy.’^50^

Some patients readmitted themselves following their discharge. George Longmore was discharged in 1880. In 1884, two months after the death of his sister, Longmore felt ‘that he was not fit to take care of himself’ and he returned to Broadmoor.^51^ He told his brother: ‘Me coming to Broadmoor I know has been a sad blow to your feelings. I am sorry for causing you so much pain… I shall be made as comfortable as can be while here.’^52^ Other patients wanted to return to Broadmoor (or stay there) for other reasons. In 1887 John Wendover was transferred, at his own request, to the Holloway Sanatorium, a private asylum in Surrey where entertainment, culture and leisure were promoted into the 1890s.^53^ Wendover’s depiction of his time at Holloway suggests that he preferred life at Broadmoor. In October 1891 he wrote to Nicolson:

to ask your advice concerning what I consider unfair treatment I am receiving at the hands of Dr Phillips… I am made a close prisoner, placed in the no 2 gallery to associate with epileptics and dementia cases, I do not quite see the object whether it is to drive me out of my mind or to force me to leave… I feel that Dr P has always openly shown an antipathy towards me… if some better arrangement… cannot be made I think I might as well be interred at Broadmoor… as I feel that the treatment I am receiving at present will make me seriously ill.^54^

Wendover’s depiction of Phillips as authoritarian and Holloway as prison-like is in contrast to his depiction of Nicolson as understanding and kind, and of Broadmoor as a place of refuge and treatment. Other patients’ representations of treatment at Broadmoor also remained positive into the 1890s. Henry Dodwell told another patient that he was feeling ‘giddy’, ‘queer’ and neglected by his family, his only consolation being the asylum band: ‘I am very glad and cheered when I hear the flute.’^55^ Historians have debated whether nineteenth-century asylums cured the mad or acted merely as a means of removing the deviant and dangerous from the community. The fact that some of Broadmoor’s patients commented positively upon the asylum’s regime in reference to their mental health, and that others readmitted themselves, is important. As Oonagh Walsh argued in her study of Irish asylums, such evidence ‘raises the possibility that patients did in fact regard the asylum as a means of recovery’.^56^ Of course, it may not always have been a question of voluntary choice but one of an inner psychological compulsion. Patients may have become pathologically dependent on the asylum’s routines and on the care of its staff, or unable to cope with life away from the institution. For some, Broadmoor simply offered respite from the world, as was also the case at other asylums.^57^ Familial relationships were often fraught, with some patients reluctant to leave Broadmoor on these grounds.^58^ Others found re-establishing familial relationships difficult following their discharge. Mary Ann Miller, who had been discharged into her husband’s care, told Orange: ‘I would rather be under your care.’^59^ In the case of another (male) patient, it was reported: ‘There can be no doubt that… asylum life has been most beneficial in this case, where the mind was unable to sustain the strain and pressure of business anxiety.’^60^ Many men were admitted to Broadmoor suffering from work-related insanity and, once there, had the opportunity to live out a fantasy version of productive labour, cushioned from the fears and hardships of, and the competition for, work.^61^

Due to the scarcity of records, historians have found it difficult to ascertain how the poor viewed asylum treatment, but the vast number of letters that Orange and Nicolson received from patients’ predominantly working-class friends and relatives help to illuminate their views of the asylum.^62^ Of course, some repeated requests for news might simply indicate desperate hope for good news rather than confidence in the treatment given, but letters were received explicitly praising Broadmoor and enquiring into the health of loved ones. A friend of patient Timothy Grundy told Nicolson:

Your aim of curing and setting free all you consciously can is a Noble and God like one… just my idea of what all asylums ought to aim at… [you] have solved the problem of how to help and relieve [patients] without… destroying their self respect.^63^

Not only does such evidence illuminate working-class perceptions of the asylum, but it also suggests that some laymen considered Broadmoor to be a curative institution or, at the very least, one within which patients would receive a good standard of care. Historians have uncovered similar responses to treatment at the Gloucester Asylum and the York Retreat and thus, alongside these other scholarly investigations, the Broadmoor letters help to qualify Andrew Scull’s suggestion that asylum patients’ relatives were sceptical about the level of treatment and care they would receive.^64^

Broadmoor also impressed some medical men. Whilst he was superintendent, Orange invited authorities on crime and insanity to the asylum, including, in August 1881, members of the International Medical Congress. Following his visit, French physician, Dr Motet, told the French Government: ‘We have returned from Broadmoor satisfied at having found the realisation of an idea that has always appeared to us to be right.’^65^ Delegates from the French Senate visited Broadmoor in 1883 and, as former Broadmoor superintendent David Nicolson later noted, reported:

Despite the fine exterior appearance, the liberality of the accommodation, and the exceptional care bestowed upon the dietary, there is no unnecessary extravagance… one recognises at once… the unexpected spectacle of good order, tranquility, and perfect discipline.^66^

The following year, as highlighted by British alienists T.W. McDowall and Daniel Hack Tuke, they hoped ‘that France will soon have its Broadmoor also’.^67^ It should be acknowledged that British alienists quoted such opinions. As with the asylum’s annual reports, the potential for promoting British medical practice and opinion existed here, but so too did other, less self-congratulatory motivations. Nicolson quoted the Senate’s report in his obituary of William Orange to highlight Orange’s dedication and success at Broadmoor. Of course, as a close friend and colleague of Orange, and a strong proponent of the value of the criminal asylum, Nicolson was unlikely to have written anything uncomplimentary.^68^ Nevertheless, the report suggests that Broadmoor gained a reputation as a world-class institution within which patients were treated properly, and this is noteworthy for two reasons. First, it marks a change from the beginning of the nineteenth century when no attempt to treat criminal lunatics was made in Britain. Second, it shows that some medical men, having seen the workings of Broadmoor, considered the institution to be curative during a time commonly associated (by both contemporaries and historians) with pessimism and the incurable nature of insanity.^69^

Moral value was ascribed to work in asylums to relieve idleness and prevent patients dwelling on their condition.^70^ Asylum patients’ work tended to be gendered and, at Broadmoor, both sexes worked in the laundry and more men than women were employed in the kitchens.^71^ On the other hand, only males worked on the farm or in the gardens, and needle and fancy work were female occupations. As at some county asylums, male patients were encouraged to work at their trade, or invited to acquire a skill.^72^ Unlike at these other institutions, however, Broadmoor’s patients were not pressured to work; in 1876 Orange described coercion into employment as ‘cruel and unsuccessful’.^73^ Although some patients happily worked, others outright refused. Charles Lanham told an attendant that he was ‘not inclined to be industrious’ because ‘he could not make a fortune out of hard work’.^74^ Such evidence, and the fact that employment was not compulsory, casts doubt upon Michel Foucault’s description of asylum employment as

a constraining power superior to all forms of physical coercion, in that the regularity of the hours, the requirements of attention, the obligation to produce a result detach the sufferer from a liberty of mind… and engage him in a system of responsibilities.^75^

It instead supports Anne Digby’s suggestion that the extent to which asylum employment was coercive is questionable.^76^

Foucault argued that asylums were receptacles for the confinement of social outcasts.^77^ That Broadmoor patients were considered both criminal and insane may suggest, as former Broadmoor psychiatrist Harvey Gordon recently declared, that they were ‘stigmatised as the psychiatric leper of society’ and thus should be hidden from view.^78^ Yet contemporaries, including Orange and Nicolson, did not consider Broadmoor’s (Queen’s pleasure) patients to be criminals or social outcasts; they were simply ill individuals who required treatment.^79^ Moreover, an examination of discharges from the asylum questions the extent to which Broadmoor may be considered an institution of control. Following Andrew Scull’s suggestion that moral treatment was designed ‘to *transform* the lunatic’ into ‘the bourgeois ideal of the rational individual’, historians have shown that asylum patients were expected to display ‘normative standards of behaviour’, which were usually gendered, before they were discharged.^80^ At Broadmoor, the medical authorities encouraged traits including industry, temperance, self-control, rationality and decorum.^81^ However, while patients were theoretically ineligible for release until they had demonstrated ‘sane’ (often gendered) attributes, this was not always the case. Some patients were refused liberation because they were considered either a threat or a potential nuisance to society, but other reportedly idle and mentally defective (male) patients were discharged.^82^ Thus, unlike at some other asylums, recovery was not always ‘defined as having taken place when… patients could fend for themselves in the labour market’.^83^ Therefore, I suggest that, as with bourgeois asylums, an argument of social control is difficult to apply to Broadmoor, an institution with a predominantly working-class population.^84^

Historians (and contemporaries) agree that moral treatment failed. Asylums had limited resources with which to implement their ‘extravagant’ claims of a cure and, from the 1860s, the existence of long-term chronic lunatics led to rapid growth in the county asylum population.^85^ Increasing patient numbers led to the belief, expressed by alienists, including Henry Maudsley and Thomas Clouston, that insanity was hereditary, the result of degeneration and incurable.^86^ Such ideas contributed to the reintroduction of mechanical restraint and seclusion in asylums.^87^ Simultaneously to these changes, Meyer, and even more so Orange and Nicolson, eminent alienists who would have been aware that changes were occurring in asylum practice, and why, expressed the belief that Broadmoor was a curative institution and that its patients should (and could) be treated with kindness.^88^ Their reports reflected this, but so too did patients’ correspondence. Nicolson, although a colleague of Maudsley and supportive of his ideas generally, did not support the assumption that insanity was inevitable or terminal: ‘the broad fact [is] that all insanity is to some extent preventable.’^89^ Moreover, in 1888, he declared that he had ‘no intention of breaking our long record by the introduction of mechanical restraint’, as was happening at other asylums.^90^

It is not surprising that Broadmoor’s regime remained optimistic in a climate of increasing pessimism. Orange and Nicolson were not subject to the same pressures that some county asylum superintendents had to endure at this time, such as overcrowding. Unlike at other asylums, Broadmoor’s patient numbers were manageable, and moral treatment worked best at small institutions where the superintendent could take a paternal approach. Moral treatment was also initially applied towards insane convicts at Broadmoor. The following section examines the evolution of the treatment of insane convicts at the asylum and demonstrates how patient classifications eventually came to affect treatment.

## 2 Treating Insane Convicts

Between 1863 and 1900, thirty-six per cent of women and fifty-one per cent of men committed into Broadmoor were insane convicts, transferred to the asylum from prison.^91^ Most were male recidivists, and they are the focus of this section.^92^ Under John Meyer, and upon the recommendation of the Lunacy Commissioners who believed ‘it is the matter of the gravest doubt whether insane persons of the criminal class… should be treated differently from other patients’, convicts and Queen’s pleasure patients were housed side by side.^93^ Moreover, the language of moral treatment, along with practical efforts to treat patients with kindness, patience and various amusements, was applied to both classes. This changed when Orange became superintendent in 1870. Orange took starkly different approaches towards insane convicts and Queen’s pleasure patients; so too did Nicolson. From the early 1870s onwards, Broadmoor’s criminal class were considered physically and mentally degenerate, and incurable. Although this shift might appear to oppose the argument that Broadmoor’s regime remained optimistic, it is not as simple as it might seem, as I will now demonstrate.

It is perhaps no coincidence that the stance towards convicts at Broadmoor corresponded to the increasingly damning image of the male criminal that emerged in scientific and legal discourse during the late 1860s and early 1870s, when representations of recidivists became couched in the language of science, sociology, and anthropology.^94^ Some late Victorian alienists and criminologists associated recidivism with mental illness, and it was also considered to be incurable.^95^ To Maudsley, the mere existence of the recidivist proved the existence of a ‘tyranny of organization’: ‘they go criminal as the mad go mad, because they cannot help it’.^96^ In addition, recidivists belonged to the so-called ‘underclass’, and they were deficient in self-control, insubordinate, and unable to ‘apply themselves to steady and systematic work’.^97^ The assumed inherited and incurable nature of their condition meant that no time in prison would reform them.^98^

Such representations were echoed at Broadmoor, where the crimes that recidivists had committed served to illustrate their inability and unwillingness to function in society: burglary, forgery, fraud and embezzlement, housebreaking, receiving stolen goods and robbery.^99^ Moreover, recidivists’ incurable mental weakness, cunning, and disruptive nature posed issues for their treatment. In theory, leisure, religious and secular teaching, and employment were initially expected to treat both classes of patient but, in practice, this proved difficult. In the early 1870s, the reportedly unteachable and disruptive nature of recidivists brought an end to secular teaching.^100^ In contrast to the positive comments he made about female patients’ susceptibility to religious teaching, in 1869 Burt warned the Council of Supervision: ‘There is a danger of desecrating sacred service if disorderly [convict] patients should be admitted to chapel as part of medical treatment.’^101^ Convicts were eventually prohibited from attending chapel. Recidivist William Heaps arrived for his second stint at Broadmoor in 1888 and discovered that things had changed since his discharge in 1874: ‘I found it hard when told… that the convicts “could not be allowed to go to chapel”.’ He demanded ‘a removal back to prison where I shall be allowed… to follow my religion’.^102^

Broadmoor’s convicts were represented as abusive, dirty, childish, and manipulative, and their behaviour was deemed contagious. In 1874, it was declared in the *British Medical Journal*: ‘These lunatic convicts contaminate and offend… all the other patients with whom they come into contact.’^103^ Echoing such observations, Orange believed that Queen’s pleasure patients would be ‘contaminated by the degraded habits and conversation of the criminal class’ and, upon becoming superintendent, he increased the hours convicts spent in seclusion and separated the two classes.^104^ Queen’s pleasure patients, many of whom reportedly ‘expressed their strong disapproval of having to associate with convicts’, might have welcomed such changes, but some convicts viewed Orange’s regime in terms of punishment rather than treatment.^105^ Recidivist Abraham Thompson wrote the ‘Broadmoor Prisoner’s Prayer’:

Eternal God from heaven send

Thy curses on this place

Stretch forth thine hand omnipotent

This Broadmoor-hell erase

The demon Orange Lord blot out

His minions Lord destroy

Blast with Thy all-devouring breath

These imps of devilry^106^

Thompson’s prayer highlights the conflict that existed between some convicts and the asylum and it contradicts the positive representations of Orange found in Queen’s pleasure patients’ correspondence. The prayer might have been kept as evidence of Thompson’s perceived obstinate and insane behaviour; indeed he was notoriously troublesome. Such evidence must not be disregarded because a patient was considered insane. As Sander L. Gilman stated: ‘The private worlds created by the insane in their anguish are quite real. They are expressions about the myths they cast into the world and the fears they project into it.’^107^ Regardless of whether Thompson’s feelings were justified, the poem must be viewed as a reflection of his perception of his treatment at Broadmoor. The other documents contained in Thompson’s file suggest that he was deemed irrational and disruptive. It was behaviour such as Thompson’s that justified Orange’s separation of the two classes at Broadmoor.

The separation of the classes was soon deemed insufficient. The Lunacy Commissioners reported:

The forced association of honest and well-conducted persons who, solely owing to mental disease have broken the law, with convicts… is evidently unjust, and there is every reason to believe that the successful management and treatment of both classes should be more safely and efficiently conducted in separate institutions, with different rules and modes of treatment.^108^

In 1874, the Home Office, influenced by the Lunacy Commissioners and Orange, decided to incarcerate convicts at Woking Prison instead of Broadmoor.^109^ Intellectual distinctions between different types of insanity and criminality thus mapped onto logistical, practical and physical acts of segregation. Removing convicts from Broadmoor better enabled the treatment and care of Queen’s pleasure patients who, the Lunacy Commissioners reported, became ‘more manageable’.^110^ They continued to make such observations as the years passed, and thus all but directly confirming Orange’s contention that the two classes could not be treated at the same institution.^111^ This separation was not to last, however. Doubts about the legality of housing insane convicts in a prison rather than a legally recognised criminal asylum, and the belief that Woking was unsuitable for treatment, led to the construction of a new block at Broadmoor specifically for convicts.^112^ They returned to the asylum, under Nicolson’s charge, in 1888.^113^ The asylum reportedly witnessed an immediate increase in the proportion of disruptive patients and, in 1889, a convict attacked Nicolson in the airing court.^114^ As a result, the convict airing courts were asphalted.^115^ The block thus became increasingly gloomy and prison-like simultaneously to the authorities promoting bright and cheery surroundings for Queen’s pleasure patients, as previously discussed.^116^

Like Orange, under whom he had been deputy superintendent, Nicolson noted the ‘contagious evil influence’ of recidivists.^117^ Moreover, he had previously pointed out the peculiarity of the criminal mind in numerous articles written while a Prison Medical Officer.^118^ While Nicolson did not support the assumption that insanity was unavoidable, as discussed earlier, he did draw from Maudsley when he advised that the existence of an ‘unavoidable “tyranny of (criminal) organisation”’ must be considered.^119^ Nicolson’s views seemingly had an impact on his approach at Broadmoor. His annual reports highlight his damning perception of the criminal, and convicts’ letters suggest bitterness towards the approach of Nicolson and the chaplain under his charge. William Heaps complained: ‘I was told before I came here that Nicolson would treat all alike, well such a prediction is… untrue.’^120^ While reading was considered therapeutic, Heaps claimed he was denied access to the same material offered to Queen’s pleasure patients: ‘I asked the chaplain for a vol. of PE. He informed me that there was only one vol. in the block. A pleasure man… asked him for one and the chaplain sent the gentleman two vols.’^121^

The separation of Broadmoor’s two classes of patient reflected an increasingly antagonistic discourse regarding the criminal that began to emerge during the latter half of the nineteenth century, and it suggests two things. First, it indicates how broader societal concerns from the 1880s regarding the existence of a residuum were reflected at the institution, as Peter Bartlett has shown also happened elsewhere.^122^ The criminal man, whose innate idle and cunning nature was deemed contagious and a threat to the functioning of society, was also deemed a threat to the recovery of previously industrious men (as Queen’s pleasure patients were deemed to be) at Broadmoor, as well as to the general running of the asylum. The irredeemable recidivist was thus separated from hard-working individuals in both social and anthropological discourse and at the asylum. Second, Orange and Nicolson not only separated the insane and the criminal physically, but their publications and reports indicate that both men viewed the criminal and the insane to be two distinct groups requiring different modes of treatment and confinement, something which seemingly affected the experience of some convicts at the asylum.^123^ Orange and Nicolson’s concern about the criminal class highlights their continued optimism about the potential to treat and care for the insane. To them, the presence of the criminal at Broadmoor hindered the asylum’s function as a curative institution in which the insane could receive proper supervision and care.

## 3 Psychiatric Pessimism at Broadmoor

Broadmoor was not immediately affected by broader changes in psychiatry but it did eventually succumb; albeit thirty years after historians usually suggest psychiatric pessimism took hold. No definite shift towards pessimism at Broadmoor is evident until after Richard Brayn [1896–1910] was appointed superintendent in 1896. Over the latter decades of the nineteenth century, Broadmoor’s patient population increased steadily resulting in the construction of further accommodation blocks. By the end of the century, there was space for 187 females and 481 males. Between 1893 and 1898, admissions outnumbered deaths and discharges combined by an average of seven per cent.^124^ In 1899, Brayn reported to the Home Office that there was no room for any more male patients and declared that he had no choice but to house ‘turbulent and dangerous lunatics with those of a quieter disposition’, so hindering treatment.^125^ As had happened at other asylums, it was only once patient numbers increased that Broadmoor’s staff were responsive to ideas of incurability and degeneration that had been expressed since the late 1860s. In direct contrast to Orange’s view that patients were sent to Broadmoor to be cured, Brayn told the Home Office:

There is no reason to believe that treatment will be more successful in the future… The majority of Male inmates of this Asylum are not favourable subjects for treatment. By far the greater number are chronic and incurable lunatics.^126^

Brayn, like the county asylum superintendents of the 1860s and 1870s, seemingly had little choice but to alter the treatment of patients accordingly. Unlike some county asylum superintendents, he did not reintroduce mechanical restraint, and thus marked out the asylum from some county asylums.^127^ Instead, the number of hours patients spent in seclusion soared. In 1896 and 1900, 200,000 hours of seclusion were logged, with 177,000 in 1899.^128^ These figures were in stark contrast to those under Orange: 16,893 in the year 1877–78 and 3,339 in the year 1878–79.^129^ The Lunacy Commissioners expressed their concern about Brayn’s heavy use of seclusion to the Home Secretary, who considered it to be a medical matter and refused to intervene.^130^

Brayn’s previous employment may have influenced his approach. Unlike previous superintendents, he had not been deputy superintendent of Broadmoor but had worked in the increasingly regulated Victorian prison system, within which a growing number of institutions adopted seclusion and restraint to treat and subdue criminals.^131^ However, it is unlikely that the Council of Supervision, who recommended a candidate for superintendent to the Home Office, chose Brayn because there was already a shift to pessimism at the asylum: they were an optimistic group of men. Despite increasing patient numbers, the Council opposed the changes occurring under Brayn and fought to promote Broadmoor as a curative institution. When the Home Office suggested constructing another block to reduce overcrowding in the late 1890s, the Council argued, unsuccessfully, for the construction of a new asylum because they feared an increase in patient numbers would prevent the superintendent from having the ‘intimate personal knowledge of the bodily and mental state of each individual patient’ that they felt was necessary for treatment.^132^ It is perhaps no coincidence that the Council’s objections echoed the paternal ethos of Broadmoor’s former superintendent William Orange who had previously stressed both the importance of ‘doctoring the patients’ and the curative nature of Broadmoor: between 1892 and 1904 he was a member of the Council.^133^

Writers and scholars have previously suggested that Brayn viewed Broadmoor as a prison not an asylum and that his regime was one of terror, surveillance and strict regulation within which he discouraged ‘any feeling of common humanity’ between staff and patients.^134^ The Broadmoor archive contains sources that offer a new perspective of Brayn’s regime. Away from the overcrowded male refractory blocks, which Brayn expressed concern about, a different image of Brayn, and of Broadmoor, emerges. Brayn’s approach was certainly more pessimistic than any that had preceded it, but it was not one he applied to all (Queen’s pleasure) patients. Under Brayn, the language and practice of moral treatment persisted and provisions for entertainments, which Brayn acknowledged were ‘a valuable and necessary means of treatment for the insane’, increased.^135^ Rather than keeping patients and staff apart, Brayn played cricket with them; something he observed provided ‘a fund of interest and amusement’ for all involved.^136^ The evidence suggests that while Brayn’s regime was extremely tough for some, aspects of it remained focused upon the successful treatment of insanity. This finding therefore qualifies the recent suggestion that late Victorian alienists lost confidence in the treatment they could offer.^137^ As Louise Hide noted: ‘Alienists attempted to find a compromise between sifting out and treating a minority, while managing large numbers of the chronically disordered who would spend the rest of their lives in one institution or another.’^138^

Patients’ letters further enrich and complicate the transition from positivism to pessimism at Broadmoor. Even once psychiatric pessimism had all but replaced therapeutic optimism, some patients and their families continued to represent the asylum positively. Like his predecessors, Brayn received letters thanking him for his kindness and support as well as for the (sometimes successful) treatment received.^139^ It was not the case for all patients, but the letters indicate that, even if there was no hope of a cure, Broadmoor continued to offer refuge, comfort and opportunities for companionship. Unlike what historians have suggested happened at other asylums following the onset of pessimism, Broadmoor was not an institution within which all patients were forced to endure a ‘crushing and cruel’ life sentence as a result of an incurable illness.^140^

## 4 Conclusion

The privileged access to patients’ letters that the Broadmoor records provide not only reveals how some patients and their families viewed the asylum, but also offers a new perspective on the evolution of treatment in Victorian asylums, helping to enrich our understanding of the transition from therapeutic optimism to psychiatric pessimism. These records, alongside a close reading of Broadmoor’s official records and the publications of its superintendents, indicate that the move to pessimism at the Victorian asylum was not as straightforward or all encompassing as current historiographical assumptions suggest. When it came to treating the criminal class at Broadmoor, the implementation of moral treatment was short lived. Some convicts commented negatively about the changes to their treatment and tended to represent their asylum experience in more negative terms than Queen’s pleasure patients. Where Queen’s pleasure patients were concerned, the language and practice of moral treatment continued at the asylum into the late nineteenth century. Queen’s pleasure patients’ accounts indicate that the broad shift from positivism to pessimism at the asylum had little impact on their experience, with some continuing to represent their time at Broadmoor positively: it continued to be a place of refuge, comfort and companionship even under so-called psychiatric pessimism.

## Footnotes

1.
Berkshire Record Office (BRO), D/H14/D2/2/1/1116, letter, 10 August 1883. Hereafter, all references beginning D/H14 are from the BRO.

2.
EdwardShorter, A History of Psychiatry: From the Era of the Asylum to the Age of Prozac (New York and Chichester: Wiley, 1997), 33.

3.
RogerSmith, Trial by Medicine: Insanity and Responsibility in Victorian Trials (Edinburgh: Edinburgh University Press, 1981), 22.

4.
C.M.Burnett, What Shall We Do With the Criminal Lunatics? A Letter Addressed to the Right Honourable Lord St Leonards, on the Introduction of His New Lunacy Bills (London: Highly and Son, 1853).

5.
W.C.Hood, Suggestions for the Future Provision of Criminal Lunatics (London: John Churchill, 1854), 10: 27.

6.
John CharlesBucknill, Unsoundness of the Mind in Relation to Criminal Acts 2nd edn (London: Longman, Brown, Green, Longmans & Roberts, 1857), 119.

7.
John CharlesBucknill, An Inquiry into the Proper Classification and Treatment of Criminal Lunatics: A Letter Addressed to Samuel Trehawke Kekewich (London: John Churchill, 1852), 10.

8.
*Ibid.*For Dundrum, see Pauline Prior, *Madness and Murder: Gender, Crime and Mental Disorder in Nineteenth-Century Ireland* (Dublin & Portland, OR: Irish Academic Press, 2008).

9.
‘Medico-Parliamentary’, *The Lancet*, 25 July 1857, 96; Criminal Lunatic Asylum. A Bill to Make Better Provision for the Custody and Care of Criminal Lunatics, 1860 (175) 11.811.

10.
Rules for the Guidance of the Officers of Broadmoor Criminal Lunatic Asylum(London: George E. Eyre and William Spottiswoode, 1863), 3.

11.
A second block for female patients opened in May 1867.

12.
Patients were not referred to as ‘inmates’. The term ‘convicts’ was applied only to insane convicts but they were treated as patients.

13.
Deborah Weiner, “‘This coy and secluded dwelling”: Broadmoor Asylum for the Criminally Insane’, in Leslie Topp, James E. Moran and Jonathan Andrews (eds), *Madness, Architecture and the Built Environment* (New York and London: Routledge, 2007), 131–48; Jonathan Andrews, ‘From Stack-Firing to Pyromania: Medico-Legal Concepts of Insane Arson in British, US and European Contexts, c. 1800–1913. Part 1’, *History of Psychiatry*, 21, 3 (2010), 243–60. Broadmoor’s infanticidal patients have been examined. Jonathan Andrews, ‘The boundaries of Her Majesty’s pleasure: discharging child-murderers from Broadmoor and Perth Criminal Lunatic Department c. 1860–1920’, in Mark Jackson (ed.), *Infanticide: Historical Perspectives on Child Murder and Concealment, 1550–2000* (Aldershot: Ashgate Publishing, 2002), 216–48; Jade Shepherd, “‘One of the Best Fathers Until He Went Out of His Mind”: Paternal Child-Murder, 1864–1900’, *Journal of Victorian Culture*, 18, 1 (2013), 17–35. Popular histories have focused on Broadmoor’s famous patients. For example, Nicholas Tromans, *Richard Dadd: The Artist and the Asylum* (London: Tate Publishing, 2011). Criminal lunatics at the Devon and Warwick asylums have been examined: Joseph Melling, Bill Forsythe and Richard Adair, ‘Families, communities and the legal regulation of lunacy in Victorian England: assessments of crime, violence and welfare in admissions to the Devon Asylum, 1845–1914’, in Peter Bartlett and David Wright (eds), *Outside the Walls of the Asylum: This History of Care in the Community 1750–2000* (London and New Brunswick, NJ: The Athlone Press, 1999), 153–180; Janet Saunders, ‘Magistrates and madmen: segregating the criminally insane in late-nineteenth century Warwickshire’, in Victor Bailey (ed.), *Policing and Punishment in Nineteenth Century Britain* (London: Croom Helm, 1981), 217–41.

14.
Roy Porter, ‘The Patient’s View: Doing Medical History From Below’, *Theory and Society*, 14, 2 (1985), 175–98. Alexandra Bacopoulos-Viau and Aude Fauvel, ‘The Patient’s Turn Roy Porter and Psychiatry’s Tales, Thirty Years On’, *Medical History*, 60, 1 (2016), 1–18. The continued value of examining patients’ experiences is demonstrated by the recent special edition of *Medical History*, ‘Tales from the asylum. Patient narratives and the (de)construction of psychiatry’, (ed.) by Bacopoulos-Viau and Fauvel, *ibid.*See also various essays in Thomas Knowles and Serena Trowbridge (eds), *Insanity and the Lunatic Asylum in the Nineteenth Century* (London: Pickering and Chatto, 2015).10.1017/mdh.2015.65PMC484738126651185

15.
For a thorough discussion of the literature, Bacopoulos-Viau and Fauvel, *ibid.*For work making use of patients’ letters, see Catharine Coleborne, ‘Families, Patients and Emotions: Asylums for the Insane in Colonial Australia and New Zealand, c. 1880–1910, *Social History of Medicine*, 19, 3 (2006), 425–42; Louise Hide, *Gender and Class in English Asylums, 1890–1914* (Basingstoke: Palgrave Macmillan, 2014); Charlotte Mackenzie, *Psychiatry for the Rich: A History of Ticehurst Private Asylum, 1792–1917* (London and New York: Routledge, 1992); Benoit Majerus, ‘Making Sense of the ‘Chemical Revolution’. Patients’ Voices on the Introduction of Neuroleptics in the 1950s’, *Medical History*, 60, 1 (2016), 54–66; Leonard D. Smith “‘Your Very Thankful Inmate”: Discovering the Patients of an Early County Lunatic Asylum’, *Social History of Medicine*, 21, 2 (2008), 237–52; Akihito Suzuki, *Madness at Home: the Psychiatrist, the Patient, and the Family in England, 1820–60* (University of California Press, 2006); Louise Wannell, ‘Patients’ Relatives and Psychiatric Doctors: Letter Writing in the York Retreat, 1875–1910, *Social History of Medicine*, 20, 2 (2007), 297–313. In addition to that undertaken by Coleborne, work has been done on the views of patients and their friends and families in colonial asylums. For example, Bronwyn Labrum, ‘Looking Beyond the Asylum: Gender and the Process of Committal in Auckland, 1870–1910’, *New Zealand Journal of History*, 26, 2 (1992), 125–144.

16.
JonathanAndrews, ‘Case Notes, Case Histories, and the Patients Experience of Insanity at Gartnavel Royal Asylum, Glasgow, in the Nineteenth Century’, Social History of Medicine, 11, 2 (1998), 255–281, 281.1162043010.1093/shm/11.2.255

17.
*Ibid.*, 269; Allan Beveridge, ‘Voices of the Mad: Patients’ Letters from the Royal Edinburgh Asylum, 1873–1908’, *Psychological Medicine*, 27, 4 (1997), 899–908: 900.10.1017/s003329179700490x9234467

18.
Marjorie Levine-Clark, ‘Embarrassed circumstances: gender, poverty, & insanity in the West Riding of England in the early Victorian years’, in Jonathan Andrews and Anne Digby (eds), *Sex and Seclusion, Class and Custody: Perspectives on Gender and Class in the History of British and Irish Psychiatry* (New York: Rodopi, 2004), 123–48: 126.

19.
‘This is also true of the Morningside asylum records. See, Allan Beveridge, ‘Life in the Asylum: Patients’ Letters From Morningside, 1873–1908’, History of Psychiatry, 36, 9(1998), 431–469, 461.10.1177/0957154X980090360211623612

20.
An individual suffering from partial insanity ‘suffers delusion on one point and is sane on all others’. J. Russell Reynolds, *On the Scientific Value of the Legal Tests of Insanity: A Paper Read before the Metropolitan Counties Branch of the British Medical Association* (London: J & A Churchill, 1872), 46.

21.
Some work has been done on letters of working-class patients: *Ibid.*, Coleborne, *op.**cit.* (note 15).

22.
Andrews, *op.**cit.* (note 16).

23.
. Illiteracy did not always prevent patients from communicating in writing. In 1891, illiterate patient John Cooper sent a letter to Superintendent Richard Brayn. Another patient wrote the letter on Cooper’s behalf, noting at the bottom ‘written for him by a friend’, D/H14/D2/2/1/373/5.

24.
Anne Digby, *Madness, Morality and Medicine: A Study of the York Retreat 1796–1914* (Cambridge and New York: Cambridge University Press, 1985), 8; Nancy Tomes, *A Generous Confidence: Thomas Story Kirkbride and the Art of Asylum-Keeping, 1840–83*, (Cambridge: Cambridge University Press, 1984), xi; Michel Foucault, *History of Madness*, trans. by John Murphy and Jean Khalfa (London and New York: Routledge, 2006), 485.

25.
Andrew Scull, *The Most Solitary of Afflictions: Madness and Society in Britain, 1700–1900* (New Haven and London: Yale University Press, 1993), 272. Also, Joseph Melling and Bill Forsythe, *The Politics of Madness: The State, Insanity and Society in England, 1846–1914* (London and New York: Routledge, 2006), 46–7.

26.
‘Sarah Chaney has assessed the impact the distinction between patients at Bethlem had on asylum experiences: ‘No “Sane” Person Would Have Any Idea’: Patients’ Involvement in Late Nineteenth-century British Asylum Psychiatry’, Medical History, 60, 1(2016), 37–53.2665118710.1017/mdh.2015.67PMC4847396

27.
Robert GardinerHill, A Concise History of the Entire Abolition of Mechanical Restraint in the Treatment of the Insane; and of the Introduction, Success, and Final Triumph of the Non-Restraint System: Together with a Reprint of a Lecture Delivered on the Subject in the Year 1838; and Appendices, Containing an Account of the Controversies and Claims Connected Therewith (London: Longman, Brown, Green, and Longmans, 1857).

28.
J.Clark, A Memoir of John Conolly, MD, DCL, Comprising a Sketch of the Treatment of the Insane in Europe and America (London: John Murray, 1869), 35.

29.
Rules for the Guidance of Officers, Attendants, and Servants of Broadmoor Criminal Lunatic Asylum(London: Ford and Tilt, 1869), 3.

30.
Reports of the Superintendent and Chaplain of Broadmoor Criminal Lunatic Asylum for the Year 1864(London: George E. Eyre and William Spottiswoode, 1865), 9.

31.
*Ibid.*; *Reports of the Superintendent and Chaplain of Broadmoor Criminal Lunatic Asylum with Statistical Tables, For the Year 1866* (London: George E. Eyre and William Spottiswoode, 1867), 6–7.

32.
Reports of the Superintendent and Chaplain of Broadmoor Criminal Lunatic Asylum with Statistical Tables, For the Year 1865(London: George E. Eyre and William Spottiswoode, 1866), 8.

33.
For patients who were not Church of England, Wesleyan and Roman Catholic services were provided. A rabbi also visited Broadmoor.

34.
*Reports* (1864), *op.* *cit.* (note 30), 8.

35.
Reports of the Superintendent and Chaplain of Broadmoor Criminal Lunatic Asylum for the Year 1873(London: George E. Eyre and William Spottiswoode, 1874), 8.

36.
D/H14/A2/1/1/13, Annual Report 1898, 5–6.

37.
*Reports* (1864), *op.* *cit.* (note 30), 7–8.

38.
Reports of the Superintendent and Chaplain of Broadmoor Criminal Lunatic Asylum for the Year 1876(London: George E. Eyre and William Spottiswoode, 1877), 9.

39.
Reports of the Superintendent and Chaplain of Broadmoor Criminal Lunatic Asylum for the Year 1871(London: George E. Eyre and William Spottiswoode, 1872), 7.

40.
‘Alienists put much thought into asylum location, design and décor: John Conolly’, The Construction and Government of Lunatic Asylums, and Hospitals for the Insane(London: J. Churchill, 1847), 26–44.

41.
National Archives (NA), MH 51/49, Lunacy Commissioners’ letter.

42.
D/H14/A1/1/1/1, Lunacy Commissioners’ Report (1867); Annual Report (1899), *op.**cit.* (note 36), 13.

43.
Hide, *op.**cit.* (note 15), 95.

44.
NA, MH 51/49-4074, Lunacy Commissioners’ Report.

45.
Alfred Bligh was transferred out of block two because he was ‘a bad example to others for a man of intelligence.’ D/H14/D2/2/1/1284/29, memorandum.

46.
*Reports of the Superintendent and Chaplain of Broadmoor Criminal Lunatic Asylum for the Year 1863* (London: George E. Eyre and William Spottiswoode, 1864), 3; *Reports* (1864), *op.* *cit.* (note 30), 2.

47.
Lunacy Commissioners’ Report (1864), *op.**cit.* (note 42).

48.
Jonathan Andrews has suggested that annual reports were one method of promoting and legitimising the asylum. Andrews, *op.**cit.* (note 16), 271.

49.
See for example, D/H14/D2/2/1/729/5, letter to Orange.

50.
D/H14/D2/2/2/105, letter to Lucy Thompson.

51.
D/H14/D2/2/1/12112/21, letter to the Home Office.

52.
D/H14/D2/2/1/1212/26, letter to Thomas Longmore.

53.
Anne C.Shepherd, ‘Mental Health Care and Charity for the Middling Sort: Holloway Sanatorium, 1885–1900’, in Anne Borsay and Peter Shapely (eds), Medicine, Charity and Mutual Aid: The Consumption of Health and Welfare in Britain, 1550–1950 (Ashgate Publishing Ltd., 2007), 163–182.

54.
D/H14/D2/2/1/1074/57, letter to Nicolson.

55.
D/H14/D2/2/1/936c/29, letter.

56.
Oonagh Walsh, ‘Lunatic and criminal alliances in nineteenth-century Ireland’, in Bartlett and Wright (eds), *op.**cit.* (note 13), 132–52: 149.

57.
Anne Shepherd, ‘The female patient experience in two late-nineteenth century Surrey asylums’, in Andrews and Digby (eds), *op.**cit.* (note 18), 223–48: 236: 243; Elaine Showalter, *The Female Malady: Women, Madness and English Culture, 1830–1980* (New York: Virago Press, 1987), 98; Chaney, *op.* *cit.* (note 26), 43.

58.
See, for example, the case of Arthur Ludlow, D/H14/D2/2/1/1310.

59.
D/H14/D1/2/2/146/9, letter to Orange.

60.
D/H14/D2/2/1/905/56, medical report.

61.
Shepherd, *op.**cit.* (note 13).

62.
For the social class of Broadmoor’s patients, David Nicolson, ‘The Measure of Individual and Social Responsibility in Criminal Cases’, *Journal of Mental Science*, 24 (July 1878), 249–73: 272. In her study of Ticehurst, a private asylum, Charlotte MacKenzie found that some upper- and middle-class families ‘had low expectations about the care their relatives might receive’. Mackenzie, *op.**cit.* (note 15), 98.

63.
D/H14/D2/2/1/186/21, letter to Nicolson.

64.
Andrew Scull, *The Insanity of Place, The Place of Insanity: Essays on the History of Psychiatry* (London and New York: Routledge, 2006), 23; Smith, *op.* *cit.* (note 15.); Wannell, *op.* *cit.* (note 15).

65.
‘Broadmoor Asylum’, *The Lancet*, 20 August 1881, 358.

66.
DavidNicolson, ‘Obituary: William Orange’, British Medical Journal, 13 (January 1917), 67–69: 68.

67.
T.W.McDowall and Daniel Hack Tuke, ‘French Retrospect’, Journal of Mental Science, (April 1884), 150–153: 153.

68.
‘Nicolson viewed Broadmoor as an exciting opportunity to undertake research. He hoped that studying the asylum’s patients would help ‘prevent’ criminal lunacy, benefiting society. David Nicolson, ‘The Measure of Individual and Social Responsibility in Criminal Cases’, Journal of Mental Science, 24(July 1878), 249–273: 273.

69.
S.E.D.Shortt, Victorian Lunacy: Richard M. Bucke and the Practice of Late Nineteenth-Century Psychiatry (Cambridge: Cambridge University Press, 1986), 158.

70.
W.A.F.Browne, ‘The Moral Treatment of the Insane: A Lecture’, Journal of Mental Science, 20 (October 1864), 309–337: 318.

71.
Shepherd, *op.**cit.* (note 57), 240–1; Lunacy Commissioners’ Report (1867), *op.* *cit.* (note 42).

72.
See, for example, Hide, *op.**cit.* (note 15), 114.

73.
*Reports* (1876), *op.* *cit.* (note 38), 8.

74.
D/H14/D2/2/1/836/4, note.

75.
Foucault, *op.**cit.* (note 24), 485.

76.
Digby, *op.**cit.* (note 24), 63.

77.
Foucault, *op.**cit.* (note 24), 485.

78.
HarveyGordon, Broadmoor (London: Psychology News Press, 2012), 8.

79.
DavidNicolson, ‘Presidential Address Delivered at the Fifty-Fourth Annual Meeting of the Medico-Psychological Association, Held in London, 25 and 26 July, 1895’, Journal of Mental Science, 41 (October 1895), 567–591: 577–8.

80.
Hide, *op.**cit.* (note 15), 143; Pamela Michael, ‘Class, gender and insanity in nineteenth-century Wales’, in Andrews and Digby (eds), *op.* *cit.* (note 18), 95–122: 111–5; Shepherd, *op.* *cit.* (note 53), 225.15005914

81.
Judith Knelman suggests that murderer Christiana Edmunds was committed into Broadmoor, rather than be hanged, in order to be transformed into the feminine ideal. *Twisting the Wind*: *The Murderess and the English Press* (Toronto, Buffalo & London: Toronto University Press, 1999), 141.

82.
For example, Jesse Oakely was discharged even though he was feeble minded and ‘naturally… defective.’ D/H14/D2/2/1/227/17, medical report.

83.
Jonathan Andrews and Anne Digby, ‘Introduction: gender and class in the historiography of British and Irish psychiatry’, in Andrews and Digby, *op. cit.* (note 18), 7–44: 29.15005911

84.
For social control and the bourgeois asylum, Digby. *op.**cit.* (note 24), 8; Tomes, *op.* *cit.* (note 24), xi.

85.
J.K. Walton, ‘Casting out and bringing back in Victorian England: pauper lunatics, 1840–70’, in William F. Bynum, Roy Porter and Michael Shepherd (eds), *The Anatomy of Madness: Essays in the History of Psychiatry*3 vols (London: Routledge, 1985–88), VII (1985), 132–146: 142; Catherine Cox, Hilary Marland and Sarah York, ‘Emaciated, Exhausted and Excited: The Bodies and Minds of the Irish in Nineteenth-Century Lancashire Asylums’, *Journal of Social History*, 46, 2 (2012), 500–24: 511; Steven Cherry, *Mental Health Care in Modern England: The Norfolk Lunatic Asylum/St Andrews Hospital 1810–1998* (Woodbridge: Boydell Press, 2003), 89–90.

86.
Henry Maudsley, *Body and Mind: An Inquiry Into Their Connection and Mutual Influence, Specially in Reference to Mental Disorders being the Gulstonian Lectures for 1870, delivered before the Royal College of Physicians* (London: Macmillan, 1870), 60. For Clouston, Mackenzie, *op.* *cit.* (note 15), 173.

87.
Phil Fennell, *Treatment Without Consent: Law, Psychiatry and the Treatment of Mentally Disordered People Since 1845* (London and New York: Routledge, 1996), 48; Hide, *op.* *cit.* (note 15), 133.

88.
Following Meyer’s appointment, the Council asked him to visit Broadmoor and report any alterations he believed necessary. Meyer took ‘as my guide the generally accepted views on the care and treatment of persons of unsound mind’. NA, MH 51 49, Meyer’s Report.

89.
DavidNicolson, ‘The Morbid Psychology of Criminals’, Journal of Mental Science, 21 (July 1875), 225–253: 233; Nicolson, *op.* *cit.* (note 62), 262.

90.
Reports of the Superintendent and Chaplain of Broadmoor Criminal Lunatic Asylum for the Year 1888(London: George E. Eyre and William Spottiswoode, 1889), 6.

91.
These statistics are based on an examination of the admissions registers. D/H14/D1/1/1/1, Admissions Register, 1863–71; D/H14/D1/1/1/2, Admissions Register, 1868–1900.

92.
Reports of the Superintendent and Chaplain of Broadmoor Criminal Lunatic Asylum with Statistical Tables, For the Year 1874(London: George E. Eyre and William Spottiswoode, 1875), 7.

93.
Lunacy Commissioners’ Report (1868), *op.**cit.* (note 42).

94.
For the recidivist, Barry Godfrey, David Cox and Stephen Farrall, *Serious Offenders: A Historical Study of Habitual Criminals* (Oxford: Oxford University Press, 2010). *Reports* (1874), *op.* *cit.* (note 92), 7. Representations of recidivists were gendered: Susan Broomhall and David G. Barrie, ‘Introduction’, in Susan Broomhall and David G. Barrie (eds), *A History of Police and Masculinities, 1700–2010* (Oxon: Routledge, 2012), 1–34.

95.
StephenWatson, ‘Malingerers, the ‘Weakminded’ Criminal and the ‘Moral Imbecile’: How the English Prison Medical Officer Became an Expert in Mental Deficiency, 1880–1930’, in Michael Clark and Catherine Crawford (eds), Legal Medicine in History (Cambridge: Cambridge University Press, 1994), 223–241.

96.
Maudsley quoted in Janet Saunders, ‘Quarantining the weak minded: psychiatric definitions of degeneracy and the late-Victorian asylum’, in Bynum, Porter and Shepherd (eds), *op.**cit.* (note 85), 273–97: 277.

97.
HenryMaudsley, Body and Will: Being an Essay Concerning Will in its Metaphysical, Physiological, and Pathological Aspects (New York: D. Appleton and Company, 1884), 276–277.

98.
W.D.Morrison, Crime and its Causes (London: Swan Sonnenschein & Co., 1891), 225–226.

99.
Admissions Registers, *op.**cit.* (note 91).

100.
*Reports* (1874), *op.* *cit.* (note 92), 12.

101.
D/H14/A2/1/6/1, Minutes of the Council, 4 June 1869.

102.
D/H14/D2/2/1/1363, letter to the Council.

103.
‘Criminal Lunatics and Lunatic Convicts’, British Medical Journal, 4(July 1874), 14–16: 15.

104.
*Reports* (1873), *op.* *cit.* (note 35), 12.

105.
Lunacy Commissioners’ Reports (1873 and 1874), *op.**cit.* (note 42).

106.
D/H14/D2/2/1/1699, Broadmoor Prisoner’s Prayer.

107.
Sander L.Gilman, Disease and Representation: Images of Illnesses from Madness to Aids (Ithaca and London: Cornell University Press, 1988), 99.

108.
Lunacy Commissioners quoted in *Reports* (1874), *op.* *cit.* (note 92), 8–9.

109.
NA, HO45/9525 32338/21, memorandum.

110.
D/H14/A1/1/1/2/1/1, Lunacy Commissioners’ Report (1875).

111.
D/H14/A1/1/1/2/3/4, Lunacy Commissioners’ Report (1878).

112.
NA, HO. 45.9754. A.60166, notes of the Criminal Lunacy Commission; NA, HO45/9525 32338/20, letter to the Home Office.

113.
For discussions on construction, NA, HO45/9525 32338.

114.
Reports Upon Broadmoor Criminal Lunatic Asylum, With Statistical Tables, For the Year 1889(London: Eyre and Spottiswoode, 1890), 5.

115.
Lunacy Commissioners’ Report in *Ibid.*, 9–12: 10.

116.
Annual Report (1899), *op.**cit.* (note 36), 13.

117.
*Reports* (1889), *op.* *cit.* (note 114), 5.

118.
For example, David Nicolson, ‘The Morbid Psychology of Criminals’, *Journal of Mental Science*, 20 (July 1874), 167–185. For more about Nicolson, see Neil Davie, *Tracing the Criminal: The Rise of Criminology in Britain, 1860–1918* (Oxford: Bardwell Press, 2005).

119.
Nicolson, *op.**cit.* (note 79), 233.

120.
D/H14/D2/2/1/1363, letter to the Council, 11 July 1889.

121.
*Ibid*.

122.
Peter Bartlett, ‘The asylum and the poor law: the productive alliance’, in Joseph Melling and Bill Forsythe (eds), *Insanity, Institutions and Society, 1880–1914: A Social History of Madness in Comparative Perspective* (London: Routledge, 1999), 48–67.

123.
Nicolson, *op.**cit.* (note 79), 580.

124.
NA, HO. 45.9754. A.60166/12 report on admissions and discharges, 1899.

125.
NA, HO. 45.9754. A.60166/9, letter to the Home Office.

126.
*Ibid*.

127.
Hide, *op.**cit.* (note 15), 133.

128.
RalphPartridge, Broadmoor: A History of Criminal Lunacy and its Problems (London: Chatto & Windus, 1953), 91–92.

129.
Reports of the Superintendent and Chaplain of Broadmoor Criminal Lunatic Asylum with Statistical Tables, For the Year 1880(London: George E. Eyre and William Spottiswoode, 1881), 88–95: 93.

130.
Fennell, *op.**cit.* (note 87), 59.

131.
‘For the Victorian prison system, Martin Wiener’, Reconstructing the Criminal: Culture, Law and Policy in England, 1830–1914(Cambridge: Cambridge University Press, 1990), 105–109.

132.
Letter to the Home Office, *op.**cit.* (note 125).

133.
In March 1868, Meyer recorded that Orange had complained: ‘I am not doing my duty I am not doctoring the patients.’ D/H14/A2/1/3/1, Superintendent’s Journal.

134.
Partridge, *op. cit.* (note 128), 91.

135.
Annual Report (1898), *op.**cit.* (note 36), 5–6; *Reports Upon Broadmoor Criminal Lunatic Asylum, With Statistical Tables, For the Year 1898* (London: Darling & Son Ltd, 1899), 5.

136.
Reports Upon Broadmoor Criminal Lunatic Asylum, With Statistical Tables, For the Year 1900(London: Darling & Son Ltd, 1901), 10.

137.
AndrewScull, Madness in Civilisation: A Cultural History of Insanity from the Bible to Freud, from the Madhouse to Modern Medicine (London: Thames and Hudson, 2015), 230.

138.
Hide, *op. cit.* (note 15), 39.

139.
For example, D/H14/D1/2/2/398/41, letter to Brayn.

140.
Scull, *op.**cit.* (note 137), 229.

